# Removing Heavy Metals: Cutting-Edge Strategies and Advancements in Biosorption Technology

**DOI:** 10.3390/ma17051155

**Published:** 2024-03-01

**Authors:** Katarzyna Staszak, Magdalena Regel-Rosocka

**Affiliations:** Institute of Chemical Technology and Engineering, Faculty of Chemical Technology, Poznan University of Technology, ul. Berdychowo 4, 60-965 Poznan, Poland; katarzyna.staszak@put.poznan.pl

**Keywords:** biosorption, biomaterials, heavy metals, cutting-edge strategies, wastewater, industrial effluents, nickel, cobalt, chromium, zinc, lead, copper, mercury, cadmium, arsenium

## Abstract

This article explores recent advancements and innovative strategies in biosorption technology, with a particular focus on the removal of heavy metals, such as Cu(II), Pb(II), Cr(III), Cr(VI), Zn(II), and Ni(II), and a metalloid, As(V), from various sources. Detailed information on biosorbents, including their composition, structure, and performance metrics in heavy metal sorption, is presented. Specific attention is given to the numerical values of the adsorption capacities for each metal, showcasing the efficacy of biosorbents in removing Cu (up to 96.4%), Pb (up to 95%), Cr (up to 99.9%), Zn (up to 99%), Ni (up to 93.8%), and As (up to 92.9%) from wastewater and industrial effluents. In addition, the issue of biosorbent deactivation and failure over time is highlighted as it is crucial for the successful implementation of adsorption in practical applications. Such phenomena as blockage by other cations or chemical decomposition are reported, and chemical, thermal, and microwave treatments are indicated as effective regeneration techniques. Ongoing research should focus on the development of more resilient biosorbent materials, optimizing regeneration techniques, and exploring innovative approaches to improve the long-term performance and sustainability of biosorption technologies. The analysis showed that biosorption emerges as a promising strategy for alleviating pollutants in wastewater and industrial effluents, offering a sustainable and environmentally friendly approach to addressing water pollution challenges.

## 1. Introduction

Biosorption, a process that is gaining prominence as an eco-friendly and cost-effective method, demonstrates remarkable potential in the removal of pollutants. This separation operation is defined as a physicochemical and metabolically independent process that enables certain biomasses of biological origin to accumulate heavy metals by binding them to their cellular structures based on a variety of mechanisms, including absorption, adsorption, ion exchange, surface complexation, and precipitation [1,2]. This technique involves the utilization of various biological materials, including agricultural residues such as crop residues, fruit peels, and other agricultural by-products, as well as microorganisms, algae, and fungi. Such biobased materials serve as effective sorbents and exhibit the ability to adsorb and accumulate pollutants from aqueous solutions. This process is gaining attention as a sustainable alternative to traditional physicochemical methods of wastewater treatment. It could be concluded that biosorption is part of broader bioremediation strategies. Microorganisms and plants are used to adsorb and accumulate pollutants, helping to restore the environment.

These biological approaches leverage the natural capabilities of certain organisms to sequester, transform, or immobilize contaminants in soil, water, and air. Certain bacteria and fungi have the ability to metabolize or transform pollutants into less harmful forms. For example, certain bacteria and fungi have the ability to metabolize or transform pollutants into less harmful forms and sorb the derivatives. For example, bacteria such as *Pseudomonas* and *Bacillus species* can degrade hydrocarbons and organic pollutants [3] and adsorb heavy metals and other pollutants onto their cell surfaces, and their effectiveness can be influenced by factors such as pH, temperature, and the presence of competing ions. In addition, specific bacteria, known as metal-resistant bacteria, have the ability to survive in environments containing high concentrations of heavy metals. These bacteria can accumulate metal ions on their cell surfaces, making them suitable for biosorption applications in metal-contaminated wastewater [4,5]. However, fungi, for example, white rot fungi, such as *Phanerochaete chrysosporium*, are known for their ability to break down complex organic compounds and their ability to sorb a wide range of pollutants [6,7]. The mycelial structure of fungi provides a developed surface area for biosorption. Comprised mainly of natural polymers such as chitin, cellulose, and proteins, mycelium constitutes a natural polymeric composite material with a porous structure formed by tubular filaments known as hyphae. Typically, hyphae range in diameter from 1 to 30 μm and can extend in length from a few microns to several meters, contributing to the recognition of mycelium as one of the largest living organisms on Earth [8,9]. Fungal mycelium can entrap and accumulate heavy metals, making fungi suitable for the removal of metals from aqueous solutions. Modification of fungal biomass with chitosan, a natural biopolymer derived from chitin, enhances the biosorption capacity. Chitosan-modified fungi have been used for the removal of metals, dyes, and other pollutants from wastewater [10]. Among the arguments in favour of the use of bacteria and fungi are their high species diversity and their ability to be adapted or engineered to combat specific contaminants. In addition, some microorganisms have the ability to regenerate and continue biosorption after desorption, contributing to the sustainability of the biosorption process [11]. On the other hand, the use of various biological materials, such as biosorbents, especially agricultural residues, represents a sustainable and cost-effective approach for the removal of pollutants from wastewater and industrial effluents [12]. Agricultural residues are abundant, renewable, and often considered as waste, which makes their repurposing for biosorption an environmentally friendly solution. An excellent example is found in crop residues, biomasses from crop harvesting, such as corn stalks, wheat straw, and rice husks, which are rich in cellulose and lignin [13,14,15]. These materials have been successfully used as biosorbents for heavy metals due to their structural components that offer binding sites for metal ions [14,16]. Sugarcane bagasse, the fibrous residue left after extracting juice from sugarcane, is another example. The bagasse contains cellulose and hemicellulose and has been shown to be effective in removing pollutants, such as dyes and heavy metals, from wastewater [17,18]. In addition, fruit peels are proposed as sorbents. For example, the peels of citrus fruits, such as oranges and lemons, are abundant sources of pectin and other organic compounds. Citrus peels have been used as biosorbents for the removal of heavy metals, dyes, and organic pollutants from aqueous solutions [19,20,21,22], while banana peels, known for their high cellulose and polyphenol content, have shown promise as biosorbents [23,24]. Other agricultural by-products, such as shells and bran or nut shells, have also been tested for their biosorption capacity. In addition to the fact that agricultural residues are found in large amounts and are often considered waste, making them a viable source of biosorbents, the issue of their renewability and the ease with which they can be modified to increase their biosorption capacity through methods such as chemical modification establishes that their use as biosorbents is consistent with sustainable practices and reduces dependence on non-renewable resources. It is crucial to highlight that, in a number of cases, biowaste is converted into activated charcoal and performs as a biosorbent in this altered form [14]. However, this review focuses only on native organic sorbents, specifically on biomaterials derived from living organisms such as plants, fungi, and microorganisms, excluding sorbents such as coal, fly ashes, and biochars.

Biosorption has found applications in various industries and settings due to its effectiveness in removing pollutants from wastewater and industrial effluents. Biosorption is often integrated with other water treatment technologies, such as membrane filtration and precipitation, to achieve a more comprehensive and efficient pollutant removal. For example, biosorption is used to treat wastewater from the textile industry, where it helps to remove various pollutants, including dyes and pigments [25,26,27,28]. The versatility of biosorbents makes biosorption a valuable tool for addressing the complex nature of pollutants generated in textile processes. Furthermore, the leather industry, known for producing effluents containing chromium and dyes from the tanning process, benefits from biosorption as a means of effectively removing these pollutants [29]. There are also examples in which biosorption has been explored to treat municipal wastewater to remove pollutants, such as organic compounds, nutrients, and heavy metals [30,31,32]. It can complement or serve as an alternative to conventional treatment methods [33,34]. Biosorption can also be applied to remove excess nutrients, such as phosphorus and nitrogen, from agricultural runoff, helping to prevent eutrophication in water bodies [35]. Biosorption is also proposed to remove heavy metals, such as lead, copper, cadmium, zinc, and cobalt [36,37,38], in typical metal industries, such as mining, electroplating, and metallurgical processes. These examples show that the application of biobased materials aligns with the overarching goal of minimizing the environmental impact of industrial activities and contributes to the sustainable management of wastewater from various industries, particularly leather manufacturing and metal processing. Its versatility and eco-friendly nature positions biosorption as a promising technology with the potential to contribute significantly to the field of environmental remediation.

Despite the variety of the literature on biosorbents for heavy metal removal from model (synthetic) solutions, there is a notable scarcity of studies that evaluate these materials on real-world objects, such as wastewater and industrial effluents. This gap is significant because the practical application of biosorbents requires a thorough understanding of their performance under realistic environmental conditions. The majority of heavy metals are found in water in cationic form, whereas chromium(VI) exists as oxyanions, with specific species predominating depending on the pH level. At pH values between 0 and 6, the predominant forms are Cr_2_O_7_^2−^ and HCrO_4_^−^, while CrO_4_^2−^ becomes dominant at a pH of approximately 4.5, reaching its maximum concentration at pH values greater than 8 [39]. Furthermore, oxyanions of As(III) and As(V) are stable under a wide range of conditions in aqueous solutions. H_2_AsO_4_^−^ is predominant under oxidizing conditions at a pH less than 6.9, whereas HAsO_4_^2−^ becomes dominant at higher pH levels. In addition, H_3_AsO_4_ can be present in strongly acidic solutions, while AsO_4_^3−^ is formed under alkaline conditions [40,41]. Heavy metal speciation in wastewater affects the interactions between metal ions and biosorbents, but also the presence in real-world samples of complex matrices with coexisting ions, organic matter, and various contaminants that influence the biosorption process. The effectiveness of biosorbents can depend on factors such as pH, temperature, and the presence of competing ions, underscoring the importance of studying them in context-relevant environments. Investigation of biosorption from real solutions is crucial for validating their performance, optimizing the process conditions, and ensuring the practical applicability of biosorption for large-scale environmental remediation initiatives. Additionally, such studies contribute to environmental impact assessments, examining potential ecotoxicological effects and providing insight into the long-term implications of biosorbent applications. Ultimately, bridging the gap between laboratory studies and real-world applications is essential to advance the field and facilitate the responsible deployment of biosorbents in diverse environmental scenarios. Therefore, the objective of this paper is to provide a comprehensive review of the latest advances in biosorption processes specifically tailored for the removal of heavy metals from wastewater and industrial effluents (for the review methodology see Appendix A and Supplementary Materials). In recent years, a significant thrust of continuous research has been directed toward the enhancement of biosorption efficiency through the development of new and improved biosorbents, the optimization of process conditions, and the exploration of novel applications. This study explores the innovative landscape of biosorption technology, focusing on cutting-edge strategies for removing heavy metals from wastewater and industrial effluents. The paper presents novel insights into recent breakthroughs and advancements, shedding light on the evolving nature of biosorption applications for environmental remediation. We provide a comprehensive analysis, including an examination of various biosorbent systems, which highlights the novel contributions in biosorption technology for tackling heavy metal contamination in diverse industrial settings.

## 2. The Negative Impact of Heavy Metals on Human Health

Heavy metals are often defined as a group of metallic elements characterized by their high density, atomic weight, and potential toxicity to human health. While some heavy metals are essential for life in trace amounts (for example, Co is a constituent of vitamin B_12_), excessive or prolonged exposure to certain heavy metals can have severe negative impacts on various physiological systems. It is noteworthy that in the scientific literature the term “heavy metals” has been diversely defined to even include metalloids, such as the non-metals As and Se. As this has raised questions about the nomenclature of these elements, in this review, we adopt the definition proposed by Ali and Khan [42], i.e., “*heavy metals are naturally occurring metals having atomic number greater than 20 and an elemental density greater than 5 g/mL*”. Some exemplary metals, such as cadmium, chromium, nickel, cobalt, lead, copper, and zinc, have been selected from this group to consider the possibility of their biosorption. The only exception among the metals considered is a very harmful contaminant, the metalloid arsenic, the removal of which with biosorbents is also taken into account.

Table 1 demonstrates the negative influence of the selected heavy metals frequently reported in industrial effluents, as discussed in this review, on human health. It details their industrial sources and the detrimental effects they can have on organs, tissues, and overall well-being.

## 3. Development of Advanced/Modified Biosorbents

One key focus of current research is the creation and optimization of advanced biosorbents. This involves harnessing the potential of diverse biological materials, such as agricultural residues [12,13,24,58], microorganisms [5,58], algae [59,60,61], and fungi [6,62,63] (Table 2). Researchers are actively working to identify and engineer biosorbents with enhanced affinity and selectivity for heavy metals. This approach aims to maximize the efficiency of removing pollutants from aqueous solutions while minimizing the ecological impact.

Algae have recently attracted attention as efficient and sustainable biosorbents for metal removal due to their significant metal-binding capacity, relatively low cost, and widespread availability in all water sources with diverse surface physiochemical properties. These biosorbents are characterized by a large binding capacity related to the abundance of macromolecules in the walls of algal cells, for example, polysaccharides, proteins, lipids, and uronic acids, and sulfhydryl groups. The naturally dried biomass of the macro-green alga *Enteromorpha intestinalis* has been proposed for the simultaneous removal of coexisting contaminants, i.e., mixed cobalt ions and Congo red dye, from model solutions [38], and the mechanism of Co(II) sorption on the complex algal material was considered. The multipath binding mechanism of Co(II) biosorption by the surface of algal biomass engages functional groups on the cell surface (e.g., sulfhydryl, phosphate, carboxyl, thiol, and amino groups) as cell surface binding sites on which metal ions sorb through physical and/or chemical adsorption or ion exchange between metal cations and the cell surface containing cations such as K^+^, Na^+^, and Mg^2+^. It is important to highlight that among the four natural and cost-effective biosorbents, namely, macroalgae (*Fucus vesiculosus*), crab shells (*Cancer pagurus*), wood chippings, and iron-rich soil, the crab shell and macroalgae biosorbents exhibited higher sorption capacities for Cu(II) and Zn(II) from model solutions containing high concentrations of metal ions compared to commercial biochar and activated carbon [61].

Two new strains of lactic acid bacteria (LAB), namely, *Limosilactobacillus fermentum* CN-005 and *Lactobacillus fermentum* CN-011, were identified for their high sorption capacity and tolerance to Pb(II) [70]. These strains were utilized for the effective biosorption of Pb(II) from simulated wastewater (Table 2). Further investigation also showed that several other strains of lactic acid bacteria (*Lactobacillus brevis*) exhibit efficient sorption of Pb(II) from model solutions [74,75]. It was indicated that the mechanism of biosorption is realized through Pb(II) interactions with OH and -COO- functional groups on the walls of bacterial cells and leads to the formation of PbO and Pb(NO_3_)_2_.

Also, fungi are known for their ability to adsorb and accumulate heavy metals, confirming their great ecological and economic importance for the sustainability of ecosystems. This makes them potential biomaterials suitable for deployment as sorbents in various environmental and industrial applications. The large surface area provided by the fungal mycelium and spores is particularly advantageous for metal binding, making them beneficial materials for efficient metal removal [76]. For example, Co(II) was sorbed from water on three fungal biomasses: *Paecilomyces* sp., *Penicillium* sp., and *Aspergillus niger* [77]. *Paecilomyces* sp. showed the highest removal of Co(II), reaching 93% within 24 h of incubation in a model solution. Furthermore, the filamentous fungus *Paecilomyces* sp. was found to successfully remove 100% of Co(II) from naturally contaminated water and soil after 4 days of incubation. Similarly, *Penicillium* sp. and *A. niger* sorbed 96.4% during seven-day contact. However, the challenges lie in the kinetics of biosorption and the competition with other ions in the environment, rendering the application of fungi as biosorbents a complex task. Moreover, as presented in [78], it is possible to isolate the multimetal-tolerant fungus *Aspergillus* sp. for removing Zn, Fe, Se, and Ag nanoparticles, potential nanoscale metal pollutants, from aqueous solutions. Optimal biosorption conditions were determined, showing high biosorption percentages for two-day-old cells (91.7, 76.8, 52.2, and 39.3% for selenium, silver, iron, and zinc nanoparticles), pH 7 (80.4, 82, 68.1, and 38.8% for Se, Ag, Fe, and Zn-NPs), and specific contact times (10 min for Zn and Ag, 40 min for Fe and Se). The results indicated significant removal efficiencies for zinc, iron, selenium, and silver nanoparticles, with living fungal pellets outperforming dead biomass. However, dead fungal biomass may be more practical for environmental applications.

Another approach to the development of advanced biosorbents involves various modifications of native biomaterials to improve the sorption efficiency and selectivity toward the target metal ions or facilitate the separation of solid material from liquors. For instance, magnetically modified peanut husks have been proven to enhance the sorption of Pb(II) and Cd(II), as well as the separation of the sorbent. However, the strong interaction between sorbates and magnetic sorbents has been found to reduce the efficiency of desorption, hindering the regeneration of this modified biomaterial [79].

In turn, the chemical modification of potato starch powder through phosphorylation using disodium hydrogen orthophosphate has yielded positive results in the sorption of Pb(II) [80]. Native starch typically exhibits low sorption ability due to the lack of specific functional groups on its surface. Consequently, an increase in the specific surface area (from 2.25 to 6.75 m^2^/g) and average porosity (from 55.48 to 61.44 Å) of the modified biosorbent compared to the unmodified one can be attributed to these chemical modifications. In addition, a shift in pH_PZC_ from 5.64 to 2.01 was reported for phosphorylated starch, indicating a stronger electrostatic attraction between negatively charged biosorbents and Pb(II) ions. Other modified starches include succinylated starches, starch-based composites and nanoparticles, starch-based hydrogels, and cross-linked or carboxylated starches [81]. 

An improvement in the sorption capacity of Cd(II) (33.2 mg/g) and Pb(II) (116.7 mg/g) was reported for citric acid- and Fe_3_O_4_-modified sugarcane bagasse (MSB) compared to the unmodified sorbent [82]. The improvement was primarily attributed to an increase in the number of O-containing functional groups (e.g., hydroxyl and carboxyl or Fe-OH groups) and aromatic rings. Correspondingly, multiple mechanisms, such as surface complexation, electrostatic attraction, and cation–π interaction, were involved in the sorption of Cd and Pb by the modified sugarcane bagasse. Another method to enhance the sorption performance of Cu(II) (138 mg/g) by providing abundant active sites is the functionalization of cellulose surfaces with hyperbranched polyamide (HP) with a subnano 3D architecture [83].

## 4. Optimization of Process Conditions

To enhance the effectiveness of biosorption processes, researchers are studying the optimization of various operational parameters. This includes fine-tuning factors such as pH, temperature, contact time, and biomass concentration. Optimizing these conditions is crucial to achieving the highest possible removal efficiency and ensuring the practical applicability of biosorption in diverse industrial settings.

Generally, various mathematical and statistical approaches, including statistical design of experiments (DOE) or central composite design (CCD) for response surface methodology (RSM) [25,38,80], are employed to optimize biosorption conditions. It is often beneficial to use a combination of these methods to comprehensively understand interactions between different variables and their impact on biosorption efficiency and indicate optimal conditions.

Central composite design (CCD) was proposed to optimize biosorption of cobalt ions on dry biomass from *E. intestinalis* algae [38]. The desirability function predicted a maximum Co(II) removal yield of 85.35% with *E. intestinalis* under optimal conditions. These conditions included an initial pH value of 10, a biomass concentration of 1.0 g/L, an initial Co(II) concentration of 200 mg/L, and an incubation time of 20 min. Upon experimental verification, a Co(II) removal rate of 80.22% was achieved, confirming a high correlation between the experimental values and the predicted ones. This suggests that the CCD approach effectively optimized the biosorption of Co(II) on algal dry biomass, highlighting the reliability of the predicted optimal conditions. Furthermore, the Plackett–Burman design (PBD), the statistical experimental design technique used in the field of experimental design and optimization, particularly in the context of screening experiments to identify significant factors that affect a process, could be used successfully, as presented in [84]. The study revealed that the impact of various factors on Cr(VI) biosorption by *Streptomyces rochei* ANH was as follows: pH, biomass concentration, and agitation speed exhibited adverse effects on biosorption efficiency. However, factors such as incubation temperature, contact time, initial metal concentration, and cell viability showed negligible effects on metal removal.

Moreover, the incorporation of advanced computational techniques, such as artificial neural networks (ANNs) and genetic algorithms (GAs), is proving to be highly effective in further refining biosorption process optimization. ANNs, for instance, can model complex, non-linear relationships between various operational parameters and biosorption efficiency, which traditional statistical methods may not fully capture. They provide a robust framework for predicting optimal conditions even in highly variable industrial effluents. Similarly, genetic algorithms offer a unique approach by mimicking natural evolutionary processes to find the best combination of operational parameters for maximum biosorption efficiency. This technique iteratively alters a set of parameter values, akin to genetic mutation and selection, to arrive at a near-optimal solution over successive generations. By this means, GAs can efficiently search through a vast parameter space to identify the most effective biosorption conditions. For example, in a study focusing on the biosorption of heavy metals from industrial wastewater, an integrated approach using an ANN and a GA was implemented. The model efficiently predicted optimal pH, temperature, and contact time, which resulted in a significant increase in biosorption capacity, demonstrating the potential of these computational techniques in improving the efficiency and applicability of biosorption processes. For instance, these models were successfully adapted for the prediction of Pb(II) sorption using natural and treated *Ardisia compressa* K. leaves [85], while the efficacy of artificial neural network (ANN) and adaptive neuro-fuzzy inference system (ANFIS) techniques was explored for predicting the removal efficiency of heavy metal ions (lead and nickel) from the active sludge of an industrial wastewater treatment plant [86]. Experimental parameters, including the pH of the solution, contact time, initial ion concentration, and temperature, were analyzed to determine optimal values. The ANN utilized a multilayer perceptron network, while the ANFIS employed a Sugeno fuzzy model for modeling. Comparison between experimental and predicted data yielded satisfactory results, with correlation coefficients exceeding 98%, indicating high accuracy in both models, although the ANFIS showed slightly superior performance over the ANN. Optimal operating conditions were determined via optimization of the genetic algorithm, leading to acceptable sorption efficiency values when treating a real sludge sample under these conditions. These findings suggest that the proposed intelligent models serve as reliable tools for predicting pollutant sorption efficiencies.

Furthermore, researchers are exploring the use of machine learning (ML) algorithms for predictive modelling of biosorption systems. These algorithms can process large datasets, learn patterns, and make accurate predictions about biosorption outcomes under various conditions. This not only aids in understanding the complex dynamics of biosorption processes but also helps in scaling up the technology for industrial applications. For example, key parameters that affect Cr(VI) biosorption by immobilized *Pseudomonas alcaliphila*, including immobilized bacterial cells, contact time, and initial Cr concentrations, were identified using the Plackett–Burman matrix in [87]. A comparative analysis between the rotatable central composite design (RCCD) and an artificial neural network (ANN) was conducted to determine the most suitable model to maximize Cr(VI) biosorption. RCCD experimental data were used to train a feed-forward multilayer perceptron ANN algorithm, which demonstrated superior predictive accuracy compared to the RCCD in forecasting optimal wastewater treatment conditions. Scanning electron microscopy revealed the presence of shiny large particles on the bead surface post-biosorption, while energy dispersive X-ray analysis detected an additional peak of Cr(VI), confirming the role of immobilized bacteria in Cr(VI) ion biosorption.

The integration of computational and mathematical methods with experimental research is key to unlocking the full potential of biosorption technologies and their optimalization. Such multidisciplinary approaches not only enhance the efficiency of biosorption processes but also contribute to their scalability and sustainability, paving the way for their broader application in environmental remediation and resource recovery.

## 5. Exploration of Novel Applications

The versatility of biosorption is continually expanding through the exploration of novel applications. Researchers are investigating its efficacy in addressing emerging challenges and pollutants, thereby broadening the scope of its environmental applications. These include the removal of specific pollutants prevalent in various industries, which makes biosorption an adaptable and targeted solution for different environmental needs. Biosorption techniques are employed in bioremediation processes to remove contaminants, such as heavy metals and organic pollutants, from soil. Microorganisms and plants are utilized to absorb, degrade, or sequester contaminants from the soil matrix, contributing to soil remediation efforts. For example, rapid urbanization and agricultural intensification contribute significantly to the generation of municipal solid waste (MSW), necessitating economically viable innovations for reducing heavy metals to non-toxic levels. For such problems, Manna et al. [63] proposed efficient fungi isolated from sewage sludge for a biofiltration strategy, using them to remove substantial heavy metals from contaminated MSW compost. *Trichoderma viride*- and *Aspergillus flavus*-based biofilters exhibited high removal rates for Pb (>40%) and Cd (>20%), while *Aspergillus heteromorphus* was more efficient in removing Cu and Cr (20%). Biofilters based on *Trichoderma viride*, *Aspergillus heteromorphus*, *Rhizomucor pusillus*, and *Aspergillus flavus* demonstrated effectiveness in mitigating the toxicity of Zn (30%) and Ni (>30%). Differential minimum inhibitory concentrations, HM uptake, and biosorption capacities among fungi contributed to variations in biofilter efficacy. In addition, biosorption techniques are applied in air filtration systems to remove volatile organic compounds (VOCs), odors, and other pollutants from indoor and outdoor air. Biofilters containing microbial cultures or activated carbon filters enhanced with microorganisms are used to capture and degrade airborne contaminants [62]. Biosorption is also utilized in biological leaching processes for metal recovery from ores. Certain microorganisms are capable of selective binding to target metals, facilitating their extraction and recovery from mineral ores through bioleaching techniques [88].

## 6. Engineered Microorganisms and Nanomaterials

An exciting frontier in biosorption research involves the development of engineered microorganisms and nanomaterials tailored for enhanced biosorption capabilities. Through genetic modification and the incorporation of nanoscale materials, researchers aim to improve the selectivity, capacity, and overall performance of biosorbents. This cutting-edge approach holds promise for pushing the boundaries of biosorption technology and opening up new possibilities for efficient heavy metal removal.

There is a noticeable trend for the isolation of bacteria that are resistant to heavy metals from the environment. For example, a heavy metal-tolerant bacterium, *Oceanobacillus profundus* KBZ 3-2, isolated from mine waste (from the abandoned Kabwe Mine, Zambia) showed a high removal efficiency for Pb(II) (97%) and a lower efficiency for Zn(II) (54%) [89], while the Cd-resistant bacterium *Bacillus subtilis* KC6 screened from Cd-contaminated soil sampled from an abandoned open-air pyrite mine (Xingwen, Sichuan Province, China) showed a reduction in cadmium of up to 86% [90].

The genetic engineering trend is also noticeable. For example, the bacterium *Pseudomonas putida* has been genetically modified to express metal-binding peptides on its cell surface, enhancing its ability to sorb heavy metals from contaminated water. Moreover, genetic engineering techniques have been used to engineer *Escherichia coli* strains with enhanced metal-binding capabilities, making them effective biosorbents for the removal of heavy metals from wastewater. As presented in [91], these synthetic bacterial cells and magnetic nanoparticles could be used to remove Cd(II) and Pb(II) with over 90% efficiency and could be recycled by artificial magnetic fields. This bioengineering effort involves the integration of a synthetic metallothionein and a green fluorescence protein-encoding (GFP) reporting gene into these bacteria, facilitating the expression of a fusion protein aimed at heavy metal biosorption. Additionally, the use of magnetic nanoparticles (MNPs) coated with polyethylenimine (PEI) and diethylenetriaminepentaacetic acid (DTPA) is discussed as a method for enhancing the recruitment and recovery of these bacterial cells after heavy metal remediation tasks. These MNPs were designed to interact with the modified bacteria, facilitating magnetic separation and preventing the release of engineered genetic materials into the environment. Magnetic nanoparticles have emerged as a highly effective solution for the removal of metal ions from various environmental matrices. This innovative approach leverages the unique properties of NPs, specifically their magnetic characteristics, to facilitate the easy and efficient separation of metal ions from contaminated water sources.

## 7. Deactivation and Regeneration of Biosorbents

The issue of biosorbent material deactivation and failure over time poses significant challenges in the practical application of sorption processes for pollutant removal. Although biosorbents initially exhibit promising sorption capabilities (see Table 2), their long-term performance can be influenced by various factors, leading to deactivation and reduced efficacy. The majority of works on the use of sorbents, however, do not describe this problem. Biosorbents typically have a finite number of binding sites available for adsorption. Over time, these binding sites can become saturated as the biosorbent accumulates pollutants, decreasing its capacity to sorb additional contaminants. For example, as shown in the work on the removal of iron and phosphorus from a model solution by mango leaf biosorbents [92], the deactivation of biosorbents is attributed to the reversible or irreversible chemisorption of iron and phosphorous molecules to active sites. This reduces the number of sites available for the adsorption reaction. The poisoning may occur as a result of highly sorbed feed impurities, making the regeneration of poisoned materials challenging. The authors showed that the highest desorption rate, reaching 93.15%, was observed using 0.05 M HCl. However, the use of HNO_3_ with the same concentration as the eluent resulted in even more efficient desorption, reaching 94.56%. The sorption of iron and phosphorus on the regenerated biosorbent remained consistent for the first three cycles, maintaining a level of 97.38%. Subsequently, in the fourth cycle, a decrease was observed, reducing to 82.36%.

Furthermore, the surface of biosorbents may experience fouling due to the accumulation of impurities, organic matter, or other substances present in treated water. This fouling can lead to a decrease in the active surface area and, consequently, a decline in the sorption performance. For example, in the case of seawater purification, algae are typical biofouling microorganisms. In this case, it is proposed to prepare a biosorbent with antifouling properties, such as a 3D reticular antifouling sorbent based on polyethylenimine and guanidineacetic acid, for the extraction of uranium from seawater [93]. The material demonstrates high sorption and regeneration performance with a sorption capacity of 414.93 mg/g. Moreover, the new sorbent effectively inhibits the interaction between *Closterium venus* and material surfaces in antibiofouling assays. Hydrochloric acid (0.4 M) is also proposed as a regenerative agent for a biosorbent based on the brown alga *Sargassum polycystum* for the removal of cadmium and zinc from a model solution [59]. The maximum adsorption capacities (q_max_) were 105.26 mg/g and 116.2 mg/g for Cd and Zn, respectively, and the biosorbent demonstrated significant efficiency over five consecutive cycles of the sorption–desorption process using 0.4 M HCl, with a decrease in efficiency of approximately 5% over the five cycles.

Not without significance is the type of material that is used as a biosorbent. The structural integrity of biosorbent materials may degrade with time due to aging processes, affecting their sorption capabilities [94]. Structural changes, such as the degradation of cellulose in agricultural residues, can reduce the overall effectiveness of a biosorbent [95]. In the case of microorganisms used as biosorbents, the formation of biofilms on their surfaces can occur over time. Although biofilms can initially enhance sorption, they can also create a protective layer that hinders further sorption or promotes the detachment of microorganisms from the surface. For example, because living biofilms are dynamic communities of microorganisms, such that biofilm properties change over time, they affect the biosorption behavior [96]. Thus, biofilm incubation time on a geotextile was investigated in Cu(II) biosorption. The results showed that the biofilms incubated for one day exhibited the highest biosorption capacities in different Cu(II) concentrations, ranging from 4 to 119 mg/g. However, biosorption capacity decreased as biofilm development progressed, with the lowest capacities observed on day 21 of incubation (0.75 to 61 mg/g). This reduction in biosorption capacity, according to the authors’ opinion, was attributed to the dominance of a monolayer in Cu(II) biosorption. Although increasing the incubation time led to increased biofilm mass, inner layers became less active in biosorption, resulting in a decrease in the surface-to-mass ratio of biosorption. However, the biosorption capacity increased between days 21 and 28 due to the dispersion stage of biofilm development, where some parts were detached and released into the bioreactor solution. This was supported by the lower mass of biofilms harvested on day 28 compared to day 21, indicating the appearance of the dispersion stage during this period. 

Also, the sorption efficiency for heavy metals (Pb, Ni, Cd, and Hg in model solutions) using the biomass of brown algae (*Padina gymnospora*), green algae (*Cladophoropsis membranacea*), and red algae (*Hypnea hamulosa*) depends on time. The duration of contact between the biosorbent and sorbate might not directly influence biosorption capacity, but it serves as a constraining factor. With extended time, the biosorbent can exhibit its full sorption potential, showcasing its maximum capacity. At a specific duration, the sorbent achieves saturation, indicating complete occupation of its binding sites [60]. 

Another critical aspect concerning industrial effluents is the impact of relatively high concentrations of metal ions in real solutions, often reaching hundreds of milligrams to even several grams of heavy metals per liter. This leads to rapid saturation of the biosorbents with metals. Once saturation occurs, the sorption process must be interrupted, and a desorption operation followed by a subsequent washing step is necessary to regenerate the biosorbent for reuse while maintaining its sorption capacity. Overcoming biosorbent saturation is a challenge to ensure the continuous operation of waste treatment plants required by industries to decontaminate their effluents. In 2008, a patented solution for Cu(II) recovery from mining effluents was proposed, involving pretreatment of the liquor with conventional methods (e.g., precipitation by increasing pH, conventional solvent extraction, or emulsion liquid membrane extraction) to reduce high amounts of metal ions before treating the effluents with continuous biosorption on microorganisms (*Bacillus*, *Pseudomonas*, *Klebsiella*, *Enterobacter*, or mixtures of microorganisms that form biofilms isolated from the natural environment) immobilized on a fixed bed [97]. Laboratory tests showed that the efficient recovery of Cu(II) from leached tailings from an abandoned deposit in the north of Chile was possible using biomass of the red alga *Gracilaria chilensis.* The sulfuric leachate contained 224 mg/L of Cu(II) and 602 mg/L of Fe ions, prompting selective precipitation of iron hydroxide as a pretreatment step. This resulted in a pH of 1.5, with 150 mg/L Cu(II) and 200 mg/L of Fe ions in the final composition of the solution used for the biosorption, with a maximum adsorption capacity for Cu(II) of 0.311 mmmol/1 g reached by the red alga [66]. 

Moreover, biosorption processes are often sensitive to variations in environmental conditions, such as pH and temperature [98]. Fluctuations in these factors can alter the structure and functionality of the biosorbent, resulting in reduced sorption efficiency over time. This is especially important when applied to real wastewater. On the other hand, it has been pointed out that keeping biomass in a harsh environment for a long time would cause it to respond to harsh conditions, produce mutants, and probably spread genetic resistance to the next generation [99].

Certainly, various phenomena can simultaneously deactivate biosorbents. For example, brewer’s spent grain (BSG) exhibited an affinity for heavy metal cations in the following sequence: Mn(II) ≈ Zn(II) < Ni(II) < Cd(II) < Cu(II) < Pb(II). However, it was observed that the functional groups of BSG lose their effectiveness in successive sorption–desorption cycles either due to blockage by other cations or chemical decomposition resulting from repeated contact with a 0.1 M HCl solution. Following each sorption–desorption cycle, a 5–10% decline in Cu(II) removal was indicated, ultimately resulting in a complete loss of sorption properties after the sixteenth cycle [15]. 

Although reusability is a desirable characteristic of biosorbents, challenges may arise in the desorption and regeneration processes. Over successive cycles, biosorbents may lose their effectiveness, and attempts to regenerate them may be insufficient in restoring their initial sorption capacity. Implementing effective regeneration techniques, such as chemical treatment [69], can help mitigate the deactivation of biosorbents and extend their operational lifespan. Among the proposed solutions are modifications of biosorbent materials through physical or chemical means. Such solutions can enhance their resistance to fouling and environmental changes, improving their long-term stability. Regeneration concerns depend both on the material from which the sorbent is made and on the reagents that are used in the regeneration process. For example, the regeneration of a modified biosorbent, PD-Fe_3_O_4_@carboxymethyl chitosan (PD stands for poly(methacryloxyethyltrimethyl ammonium chloride), after Cu(II) and Cr(VI) sorption indicates a noticeable decrease in sorption performance during the initial recycling cycle, followed by minimal changes in subsequent regeneration cycles. This decline may stem from challenges related to the regeneration of groups located at the base polymer chain. Interestingly, the desorption performance improved significantly when a mixture of NaOH and NaCl solutions was used compared to a single NaOH solution. The recovery efficiency of the mixed solution reached 83.7%, with a corresponding adsorption capacity of 138.3 mg/g. This improvement could be attributed to increased competition between Cl^−^ and Cr(VI) for adsorption sites on the PD-Fe_3_O_4_@CCS surface, confirming that electrostatic interaction and ion exchange serve as primary driving forces for Cr(VI) adsorption [100]. Also, studies of the regeneration of the chitosan-based biosorbent with Fe_3_O_4_ NPs have emphasized both the decrease in arsenic sorption capacity and the possibility of regeneration for up to five cycles [101]. The results indicated a gradual decrease in the removal efficiency from 99.5% to 95.0% for As(V) and from 99.0% to 92.5% for As(III). Regeneration of the sorbent was efficiently achieved using a 0.1 M NaOH solution, which neutralized the surface groups of the chitosan, weakening the bonding forces between the sorbent surface and the arsenic species, thereby facilitating the release of arsenic species and the regeneration of the biosorbent for subsequent reuse. Moreover, combining biosorption with complementary technologies, such as membrane filtration or flotation and sedimentation, can create hybrid systems that address the limitations of biosorbent deactivation [58]. It was reported that a solution of NaOH is the most effective regenerating agent for desorbing Pb(II) from a low-cost biosorbent, such as *Citrus grandis* (Pomelo) leaves [22]. Furthermore, it enhanced the sorption capacity for up to four sorption–desorption cycles. The ability to desorb and regenerate can be attributed to the removal of wax and fats by the alkaline solution, which results in the exposure of surface functional groups and their deprotonation, thereby increasing the electrostatic attraction between the negatively charged surface of pomelo leaves and Pb(II) ions. 

Understanding and addressing the issue of biosorbent material deactivation and failure over time is crucial for the successful implementation of sorption in practical applications. Ongoing research should focus on the development of more resilient biosorbent materials, optimizing regeneration techniques, and exploring innovative approaches to improve the long-term performance and sustainability of biosorption technologies. An exemplary illustration of this approach has been demonstrated in the development of biosorption of Zn(II) from industrial effluents using *F. vesiculosus* from the laboratory scale to a pilot plant [73] (see Table 2). Pilot tests with wastewaters in the sorption–desorption regeneration cycle indicated that the treatment of diluted electroplating effluents originating from a spent zinc bath (1–100 mg/L Zn(II)) containing traces of other metals led to an increase in the column service time.

Most of the reported biosorption research is focused on the removal of heavy metals from model solutions. However, there are a few studies exploring the application of biosorbents in real systems. Table 2 provides examples of the removal of Cu(II), Pb(II), Cr(VI), Cu(II), Ni(II), and Zn(II) from actual industrial effluents.

**Figure 1:**
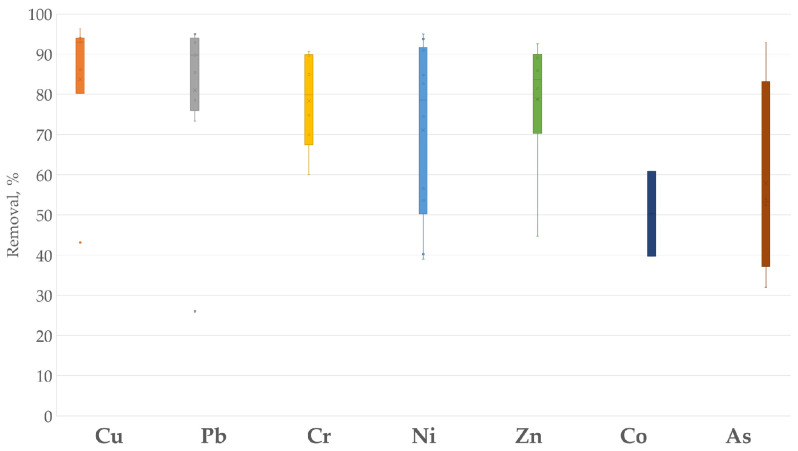
Comparison of removal of heavy metals with biosorbents.

Based on Table 2, a visualization capturing the key findings and providing a comparison of percentage removal values among different metals, expressing sorption efficiency, is shown in Figure 1. Cd(II) removal is not shown in the figure, as there was only one percentage removal value found in the literature. 

The visualization of adsorption efficiency demonstrates that various native organic biosorbents can remove metal ions such as Cu(II), Pb(II), Cr(III), and Cr(VI) from industrial effluents the most effectively (Figure 1). In spite of the efficient removal of the variety of heavy metals from industrial wastewater, to the best of our knowledge, there are neither working pilot nor industrial plants using native organic biosorbents. 

## 8. Conclusions

Biosorption has emerged as a promising strategy for mitigating pollutants in wastewater and industrial effluents, providing a sustainable and environmentally friendly solution to water pollution challenges. Ongoing research shows some pathways in this field to develop future technological advancements to enhance the effectiveness and applicability of biosorption in the future. While research on the utilization of biomaterials has experienced growth in recent years, much of it still revolves around model solutions. Furthermore, from an industrial perspective, untreated biomass does not yet appear to be considered as an attractive sorbent, unlike activated carbon or biochars derived from various biomass sources, which are commercially produced and could serve as alternatives to synthetic sorbents. Although the efficacy of metal recovery with activated carbon has been demonstrated to be highly efficient, its high cost and challenging regeneration present obstacles for treating large volumes of effluents or very diluted solutions. In such instances, native organic biosorbents are likely to be proposed as an alternative solution for effluent treatment. Certain types of algae, such as brown algae (e.g., *Fucus vesiculosus*), stand out as promising biosorbents. Algae have demonstrated significant potential due to their natural abundance, developed surface area, and ability to accumulate metals and other pollutants effectively. Additionally, agricultural by-products like rice husks, coconut shells, and sugarcane bagasse have shown promise as biosorbents due to their porous structures and chemical compositions conducive to adsorption processes.

A critical aspect involves optimizing various parameters, such as pH, temperature, biomass concentration, and contact time, to enhance biosorption efficiency, especially for the treatment of real industrial wastes that are challenging due to their complex compositions. Additionally, the regeneration of biosorbents poses a challenge due to potential damage during desorption processes or energy-intensive procedures and also due to the need to ensure the continuous operation of plants. Looking ahead, future perspectives involve ongoing research and development efforts aimed at refining biosorption processes, improving selectivity, and innovating new biosorbents. Furthermore, the integration of biosorption with complementary treatment technologies holds promise for achieving comprehensive and efficient wastewater treatment systems. The incorporation of emerging technologies, such as artificial intelligence and nanomaterials, has the potential to revolutionize biosorption techniques, providing more precise and efficient pollutant removal. Collaboration among academia, industry, and government agencies will be crucial in fully realizing the potential of biosorption to address modern environmental challenges.

## Figures and Tables

**Table 1:** The influence of the selected heavy metals on human health.

Metal	Industrial Sources	Negative Effect on Human Health	Ref.
Arsenic	Mining, smelting, pesticide manufacturing, wood preservatives	Skin lesions, cardiovascular diseases, neurotoxicity, developmental effects, diabetes, cancers (skin, lung, bladder, liver, kidney)	[43,44]
Cadmium	Metallurgical, electroplating, mining industry, manufacturing of paintings	Lung and kidney diseases; breast, lung, prostate, nasopharynx, pancreas, and kidney cancers; fetal growth restriction; skeletal damage	[45,46,47]
Cobalt	Electroplating, metallurgy, mining, superalloys “resistant to corrosion and wear”, manufacturing industries, rechargeable battery electrodes, nuclear power plants, petrochemicals, electronics, paints and pigments, chemical industry	Damage to liver, heart failure, asthma, allergy, bone defects, hair loss, low blood pressure, nervous system disorders, reduced thyroid activity (goiter), vomiting, genotoxicity, risk for cancer	[38,48]
Copper	Electroplating, metallurgical industry	Kidney damage, anemia	[49]
Chromium (especially Cr(VI))	Electroplating, tanning and mining industry	Mutagenic and carcinogenic effects, kidney dysfunction, lung cancer, and critical health impacts like diarrhea, ulcers, and damage to blood cells	[47,50,51]
Lead	Metallurgical, electroplating, metal finishing industries, manufacturing of paints, storage batteries, petroleum refining and drainage from ore mines	Risk of lung, stomach, and bladder cancer; damage to the kidney, nervous system, reproductive system, liver, and brain; causing sickness during pregnancy, Pb can also hamper fetal growth in the early stage	[45,52,53]
Mercury	Metal smelting, coal production, waste disposal, and chemical synthesis	Neurotoxicity and nephrotoxicity, cognitive impairment in children and fetal abnormalities	[45,54]
Nickel	Alloy production, electroplating, production of nickel–cadmium batteries	Allergy, cardiovascular and kidney diseases, lung fibrosis, lung and nasal cancer	[47,55]
Zinc	Electroplating, hot-dip galvanizing, metallurgy, production of batteries, pigments	Disorders to the immune system, prostate problems, diabetes and macular degeneration	[56,57]

**Table 2:** Examples of biosorbent systems applied for heavy metal removal from industrial effluents.

Metal/Sorbent	Source	Result	Ref.
**As(III)** Fungal isolates, *APR-1* (*Aspergillus niger*) and *APR-2* (*Aspergillus* spp.), immobilized on Luffa aegyptiaca (sponge gourd) (an agro-waste as biosorbent)	Industrial sewage from different regions of Davangere, Davangere District, India Conc. in mg/L 17,995.87 (As)	R in %: 53.94 and 52.54 for *APR-1* and *APR-2* No results: regeneration, the possibility of reuse, or further treatment of the biomass	[44]
**As(V)** Chemically pretreated (NaOH) unshelled *Moringa oleifera* seeds	Cassava wastewater, Nsukka, India Conc. in mg/L: 1.81–5.42 (As), 0–0.05 (Pb), 0.06–0.78 (Mn), 0.31–0.82 (Ni), 0.35–0.61 (Zn), 0.03–0.06 (Cu), 0.09–0.35 (Cd), 0.06–0.35 (Cr)	R in %: 92.9 for optimal conditions: pH 4.0, contact time of 30 min, and dosage of 2 g	[64]
**Cr(VI)** ***Aspergillus niger*:** cleaned with distilled water, boiled with 0.5 M NaOH for 15 min, rinsed with deionized water, dried at 60 °C for 24 h, and ground	Mining wastewater was recovered from a mine pit located in Abaja community, Ebonyi State, Nigeria Conc. in mg/L: 1.445 (Cr(VI)), 0.381 (Cr(III)), 0.537(Fe(III)), 0.840 (PO_4_^3−^), 0.296 (SO_4_^2−^), 0.01 (Cl^−^); pH 6, relative density 1.09, conductivity 18.7∙10^−6^ S/m, turbidity 3.92 NTU, TSS 0.12 mg/L, TDS 93.5 mg/L	q_max_: 0.0574 mg/g Conditions: 5 h contact time, biosorbent dosage 2.8 g, 200 rpm agitation speed No results: regeneration or the possibility of reuse	[37]
**Cr(III)** **Cladodes of *Oputinia ficus-inida* var. ‘Orelha de elefante’:** washed with water, cut into pieces with dimensions of 3 cm × 3 cm × 1 cm, then dried at 55 °C for 72 h and ground	Tannery stabilization pond in Brazil Conc. of Cr_2_O_3_ in g/L: 1.55–1.72, pH 7.25	R in %: 74.8 and 84.9 using 2 and 4 g of biomass, respectively q_max_: 611.49 mg/g Conditions: 60 min, without correction of pH No results: regeneration or the possibility of reuse	[29]
**Cr(VI), Ni(II)** ***Platanus orientalis* bark:** washed with water, dried in sun for 3 days, ground to a fine powder, and dried in oven at 100 °C for 24 h **Modification of sorbent:** acid activation by 0.4 M HNO_3_ and ddH_2_O for 24 h (to increase the surface area and to prevent the elution of tannin compounds)	Plating industry, Tehran, Iran Conc. in mg/L: 556.5 (Ni), 46.7 (Fe), 86.39 (Cr(VI)), 2 (Cu), 0.68 (Ag), 0.48 (Al), 0.47 (Sn), 0.44 (Pb), 0.35 (Ba), 0.18 (Sb), Hg (0.02), <0.01 (As, Bi, Co, Cd, Mo)	R in %: 89.6 and 90.7 (Cr, with non-modified and modified bark), 74.5 and 56.5 (Ni, with non-modified and modified bark) Conditions: for Cr, pH 5, 2 g/L of sorbent dosage, 5 h; for Ni, pH 3, 2 g/L sorbent dosage, 1.5 h q in mg/g: 13.42 and 19.92 (Cr, with non-modified and modified bark), 126.58 and 285.714 (Ni, with non-modified and modified bark) No results: regeneration or the possibility of reuse	[65]
**Cu(II) and Pb(II)** **Shrimp shells (without heads):** cleaned with water, dried at 70 °C for 12 h, ground, biological pigment and protein removed (mixed with 5 wt. %. NaOH and 1 wt. H_2_O_2_ for 3 days at 30 °C), and washed with water	Semiconductor electroplating wastewater (Vizianagaram, Andhra Pradesh, India) Conc. of Cu and Pb = 25–460 mg/L	Maximum efficiency in % (R): 96.4 for Cu and 89.8 for Pb Sorption capacity in mg/g (q_max_): 5.78 for Cu and 5.39 for Pb Conditions: pH 5 for Cu, pH 6 for Pb, metal conc. 20 mg/L, biosorbent dosage 0.1 g and temp. 30 °C No results: regeneration or the possibility of reuse	[36]
**Cu(II)** **Red alga *Gracilaria chilensis*** Material pretreatment: dried alga suspended in 0.2 M CaCl_2_ at pH 5 for 4 h, then washed several times with deionized water to remove excess calcium, filtered, and dried for 12 h at 60 °C	A solution obtained after leaching with 1 M H_2_SO_4_ of mining tailings from an abandoned deposit in the north of Chile and subsequent Fe precipitation Conc. in mg/L: 200 (Fe ions), 150 (Cu(II)), pH 1.5	Cu(II) q_max_ 0.311 mmol/g No data for Fe ion sorption 35% Cu(II) desorption with 0.05 M H_2_SO_4_	[66]
**Fe, Mn, Cr, As, Cd, Ni, and Pb** ***Opuntia ficus-indica* mucilage:** washed with fresh water and liquid soap before procedure of mucilage extraction (heated at 40 °C, stirred at 300 rpm for 4 h, filtered and refrigerated at 4 °C for 18 h, freeze-dried under vacuum (0.04 mbar) for 6 days)	Yautepec River, Morelos, México; sources of pollution: livestock, agricultural, recreation, public and industrial activities (automotive, food, cosmetic, pharmaceutical, colorant, textile, chemical, agrochemical, and metallurgical), ashes and gases from Popocatépetl volcano Conc. in µg/L: 4.3–14.7 (Cu), 0.2–9.5 (Cd), 3.7–13.4 (Cr), 0–44.1 (Ni), 9.3–80.8 (Pb), 7.4–19.6 (Zn), 3–405.9 (Mn), 44.5–1546 (Fe), 2–9.1 (As), pH 5.9–8.5, turbidity 0–3.4 NTU, conductivity 239–2628 µS/cm	R in %: 96 (Fe), 91 (Mn), 70 (As), 60 (Cr), 39 (Ni), 32 (Cd), 26 (Pb) No results: regeneration or the possibility of reuse	[67]
**Co(II), Ni(II), Zn(II), Cu(II)** ***Serratia marcescens* strain 16:** isolated from serpentine deposits located in Moa (Cuba)	Synthetic solution based on composition from residual liquor WL from the company Moa Nickel S. A. Cuba Conc. in mg/L: 2 (Co(II)), 25 (Ni(II)), 15 (Zn(II)), (Cu(II))	R in % after four cycles in monometallic systems: 60.9 (Co), 53.6 (Ni), 43.1 (Cu), 78.8% (Zn) R in % after four cycles in multimetallic systems: 39.7 (Co), 40.2% (Ni), 42.8% (Cu), 44.7 (Zn) The monometallic system exhibited a sorption capacity two to three times greater compared to the presence of bi-metallic and multimetallic solutions, q for monometallic solution in mg/g: 2.3 (Co), 11.4 (Ni), 8.6 (Cu), 11.9 (Zn) Conditions: contact time 2 h, biomass 0.6 g/L Desorption of metals and reuse: 0.1 M HCl for desorption (90% after 10 min), several times reduction in sorption capacity after 4 cycles	[68]
**Pb(II), Cu(II), Zn(II), Ni(II)** ***Gossypium hirsutum*** stems from the fields of Khanewal, Pakistan: dried for 15 days, washed with water, dried at 50 °C, then ground, washed with water, and dried in an oven at 60 °C. **Modification** in the solution of 0.2 M HCl and 0.2 M NaOH separately	Industrial effluents from discharge points (including textile and electric cable manufacturers; tanneries; and pesticide, pharmaceutical, and fertilizer plants) in Multan, Pakistan Conc. in mg/L: 3.7 (Pb), 3.9 (Cu), 6.3 (Zn), 2.5 (Ni)	R in %: 78.5 (Pb), 80.3 (Cu), 81.4 (Zn) and 82.6 (Ni) q in mg/g: 121.2 (Pb), 117.09 (Cu), 130.6 (Zn), 111.09 (Ni) Conditions: 0.5 g sorbent, 30 °C, pH 5.5, 30 min Regeneration using 0.1 M HNO_3,_ only from model solutions, 5 cycles, with desorption efficiency in % (92.9–85.4 (Pb), 93.2–86.1 (Cu), 92.5–85.9 (Zn), 93.8–84.8 (Ni))	[69]
**Pb(II)** **Lactic acid bacteria:** *Limosilactobacillus fermentum* CN-005, *Lactobacillus fermentum* CN-011	Simulated wastewater collected from Taihu Lake, China, and spiked with Pb(II) standard solution at five levels: 12.92, 16.17, 17.70, 20.22, and 23.94 mg/L	Average sorption efficiency of Pb(II): 73.38% with CN-011, 74.15% with CN-005 No results: the possibility of reuse or further treatment of the biomass	[70]
**Zn(II), Fe(II), Pb(II), Cu(II)** ***Arthrospira platensis*** microalgae cultivated in mining wastewater	Mining wastewater from surface and underground water in Huangshaping, Hunan Province, China	Biosorption efficiency at pH > 7.1: 93% SO_4_^2−^, 99% Fe(II), 95% Pb(II), 89% Zn(II), 94% Cu(II). No results: regeneration, the possibility of reuse, or further treatment of the biomass	[71]
**Pb(II), Ni(II)** 24 heavy **metal-resistant fungi** isolated from different industrial wastes from India (near areas of different metal-fed industries)	Sewage, sludge, and effluents collected from 23 different industrial units located at different locations in India	R in %: 93 for Pb using resistant fungi, *Aspergillus *terreus** and *Talaromyces islandicus*; 91 for Ni using *Neurospora crassa* and *Aspergillus flavus*; and 95 for Pb and Ni using the fungal consortia	[72]
**Zn(II)** Sawdust of Indian rosewood from a timber industry, Bathinda (Punjab), India **Sawdust-derived biosorbents**: after boiling (SDB), chemical modification with formaldehyde (SDF) and sulfuric acid (SDS)	A real electroplating industrial effluent (Ludhiana, Punjab, India) Conc. in mg/L: 26–46 (Zn), 0.14–1.86 (Cu), 0.05–1.76 (Ni); 37–540 ppm of sulfates, pH 1.65–5.36	Zn(II) q_eq_: 35.72 mg/g (SDB), 43.74 mg/g (SDF), 45.87 mg/g (SDS) at pH 6 Pore size of biosorbents in m^2^/g: 232.928 (SDB), 291.102 (SDF), 498.873 (SDS)	[57]
**Zn(II)** **Sugarbeet pulp and brown alga *Fucus vesiculosus*** from the northern Atlantic coast of Spain in small glass columns (2.5 cm inner diameter and 40 cm length) or *F*. *vesiculosus* in glass columns (7.5 cm inner diameter and 100 cm length)—**a pilot plant**	Continuous biosorption tests with real effluents from Industrial Goñabe (Valladolid, Spain) Conc. in mg/L: 546 (Zn), 22.9 (Fe), 10.8 (Cr), 0.129 (Cu), 0.050 (Ni), 116 (sulfate), 2520 (chloride), pH 1.45	At *F. vesiculosus* q_max_ = 0.94 mmol Zn(II)/g at pH adjusted to 5 3 consecutive cycles of continuous sorption–desorption (with 1 N HNO_3_) and regeneration with deionized water	[73]

## Data Availability

No new data were created or analyzed in this study. Data sharing is not applicable to this article.

## Supplementary Materials

The following supporting information can be downloaded at: https://doi.org/10.5281/zenodo.10702893, PRISMA 2020 flow diagram for the review; Figure S1: Cluster analysis of semantic web using VOSviewer software version 1.6.19. ref. [102] is cited in Supplmentary File.

## Appendix A. Methodology of the Bibliometric Analysis

To conduct the bibliometric analysis, the search engines employed included PubMed, Scopus, and Google Scholar. The investigation utilized specific keywords for the search process, such as combinations of biosorption with heavy metals, cobalt, nickel, lead, chromium cadmium, zinc, arsenic, wastewater, and industrial influents.

The exclusion criteria included manuscripts written in languages other than English, articles that were inaccessible, and those published before 2019 (with exceptions made for articles crucial to the presentation of foundational studies on the applications of biosorption). At the same time, to maintain originality and precision, the search specifically targeted research articles, with a deliberate exclusion of review articles. Consequently, items featuring titles containing terms such as ‘review’, ‘meta-analysis’, or ‘overview’ were omitted, except in a few instances. A PRISMA flow diagram detailing the count of identified articles, screened entries, eligibility assessments, and inclusions or exclusions in the final analysis, along with the rationales for article exclusions, is available in the Supplementary Materials.

Description of the method used for the systematic literature review:

- Planning
  **A.1 Initial idea formulation**
  Biosorption for the removal of heavy metals: addressing challenges, exploring opportunities, examining perspectives, and charting research directions.
  **A.2 PICOC**
  Population: Biosorption.
  Intervention: Use of biosorption for removal of heavy metals from wastewater.
  Comparison: The development of biosorbents for use in real sludge.
  Outcomes: Treatment support, separation efficiency, and real conditions.
  Context: Challenges and opportunities associated with the applications of biosorbents in heavy metals removal.
- Research questions
  What biosorbents can be used to remove heavy metals from wastewater?
  What is the efficiency of the biosorption process?
  For which real wastewaters can biosorption be used?
  What modifications and improvements are proposed?
  What research directions are being pursued?
  What challenges are linked to the application of biosorption processes for the removal of heavy metals?
- Digital library sources
  Google Scholar, Scopus, and PubMed.
- Inclusion and exclusion criteria
  **Inclusion**: Results obtained by searching using biosorption with heavy metals, cobalt, nickel, lead, chromium cadmium, zinc, wastewater, and industrial influents.
  **Exclusion**: Articles published before 2019 and those categorized as reviews, with a few exceptions made for studies providing fundamental insights into the issues raised.
- Quality Assessment (QA) checklist
  **E.1. Ensure that**:
  The search strategy was accurately delineated. The inclusion and exclusion criteria were suitable and clearly defined. The studies chosen aligned well with the research question.
  The selected papers demonstrated high quality, characterized by robust design and execution.
  The data were pertinent and thoroughly described. The methods used for paper inclusion were fitting and well documented. The consistency of the results was sufficiently assessed and presented.
  **E.2. Data extraction form**
  By metals: Cobalt, copper, nickel, lead, chromium cadmium, zinc, and arsenic.
  By wastewater: Metal industries: mining, electroplating, and metallurgical processes; textile industries; and the leather industry.
  **E.3. Conducting**
  Studies were gathered using the Mendeley reference management tool as a database resource.
  **E.4. Study selection and refinement**

The literature items were scrutinized for duplicates and filtered based on the exclusion and inclusion criteria outlined earlier, resulting in a curated set of articles chosen for the study.
